# Associations between arterial stiffening and brain structure, perfusion, and cognition in the Whitehall II Imaging Sub-study: A retrospective cohort study

**DOI:** 10.1371/journal.pmed.1003467

**Published:** 2020-12-29

**Authors:** Sana Suri, Scott T. Chiesa, Enikő Zsoldos, Clare E. Mackay, Nicola Filippini, Ludovica Griffanti, Abda Mahmood, Archana Singh-Manoux, Martin J. Shipley, Eric J. Brunner, Mika Kivimäki, John E. Deanfield, Klaus P. Ebmeier

**Affiliations:** 1 Department of Psychiatry, Warneford Hospital, University of Oxford, Oxford, United Kingdom; 2 Oxford Centre for Human Brain Activity, Wellcome Centre for Integrative Neuroimaging, University of Oxford, Oxford, United Kingdom; 3 Institute of Cardiovascular Science, University College London, London, United Kingdom; 4 Department of Epidemiology and Public Health, University College London, London, United Kingdom; 5 Inserm U1153, Epidemiology of Ageing and Neurodegenerative diseases, Université de Paris, Paris, France; Columbia University, UNITED STATES

## Abstract

**Background:**

Aortic stiffness is closely linked with cardiovascular diseases (CVDs), but recent studies suggest that it is also a risk factor for cognitive decline and dementia. However, the brain changes underlying this risk are unclear. We examined whether aortic stiffening during a 4-year follow-up in mid-to-late life was associated with brain structure and cognition in the Whitehall II Imaging Sub-study.

**Methods and findings:**

The Whitehall II Imaging cohort is a randomly selected subset of the ongoing Whitehall II Study, for which participants have received clinical follow-ups for 30 years, across 12 phases. Aortic pulse wave velocity (PWV) was measured in 2007–2009 (Phase 9) and at a 4-year follow-up in 2012–2013 (Phase 11). Between 2012 and 2016 (Imaging Phase), participants received a multimodal 3T brain magnetic resonance imaging (MRI) scan and cognitive tests. Participants were selected if they had no clinical diagnosis of dementia and no gross brain structural abnormalities. Voxel-based analyses were used to assess grey matter (GM) volume, white matter (WM) microstructure (fractional anisotropy (FA) and diffusivity), white matter lesions (WMLs), and cerebral blood flow (CBF). Cognitive outcomes were performance on verbal memory, semantic fluency, working memory, and executive function tests. Of 542 participants, 444 (81.9%) were men. The mean (SD) age was 63.9 (5.2) years at the baseline Phase 9 examination, 68.0 (5.2) at Phase 11, and 69.8 (5.2) at the Imaging Phase. Voxel-based analysis revealed that faster rates of aortic stiffening in mid-to-late life were associated with poor WM microstructure, viz. lower FA, higher mean, and radial diffusivity (RD) in 23.9%, 11.8%, and 22.2% of WM tracts, respectively, including the corpus callosum, corona radiata, superior longitudinal fasciculus, and corticospinal tracts. Similar voxel-wise associations were also observed with follow-up aortic stiffness. Moreover, lower mean global FA was associated with faster rates of aortic stiffening (B = −5.65, 95% CI −9.75, −1.54, Bonferroni-corrected *p* < 0.0125) and higher follow-up aortic stiffness (B = −1.12, 95% CI −1.95, −0.29, Bonferroni-corrected *p* < 0.0125). In a subset of 112 participants who received arterial spin labelling scans, faster aortic stiffening was also related to lower cerebral perfusion in 18.4% of GM, with associations surviving Bonferroni corrections in the frontal (B = −10.85, 95% CI −17.91, −3.79, *p* < 0.0125) and parietal lobes (B = −12.75, 95% CI −21.58, −3.91, *p* < 0.0125). No associations with GM volume or WMLs were observed. Further, higher baseline aortic stiffness was associated with poor semantic fluency (B = −0.47, 95% CI −0.76 to −0.18, Bonferroni-corrected *p* < 0.007) and verbal learning outcomes (B = −0.36, 95% CI −0.60 to −0.12, Bonferroni-corrected *p* < 0.007). As with all observational studies, it was not possible to infer causal associations. The generalisability of the findings may be limited by the gender imbalance, high educational attainment, survival bias, and lack of ethnic and socioeconomic diversity in this cohort.

**Conclusions:**

Our findings indicate that faster rates of aortic stiffening in mid-to-late life were associated with poor brain WM microstructural integrity and reduced cerebral perfusion, likely due to increased transmission of pulsatile energy to the delicate cerebral microvasculature. Strategies to prevent arterial stiffening prior to this point may be required to offer cognitive benefit in older age.

**Trial registration:**

ClinicalTrials.gov NCT03335696

## Abstract 2

Sana Suri and colleagues investigate differences in brain structure and connectivity associated with aortic stiffening in older adults.

## Author summary

**Why was this study done?:**

- Stiffening of large arteries such as the aorta has been closely linked to the onset of heart diseases, but recent studies show that this may also increase risk for dementia.
- Understanding the brain changes which underlie this risk could help identify strategies to prevent or delay dementia in old age.

**What did the researchers do and find?:**

- We investigated 542 community-dwelling older adults who received 2 measurements of aortic stiffness, first at approximately 64 years old and again at approximately 68 years old.
- Shortly after that, at mean age 69 years, they underwent cognitive tests and brain magnetic resonance imaging (MRI) scans to assess the size, connections, and blood supply of different brain regions.
- We observed that a faster rate of aortic stiffening during the 4 years in mid-to-late life was linked to lower blood flow and poorer markers of brain connectivity across several brain regions.
- We also found that lower memory performance in older age was most closely linked to the first measures of aortic stiffness, taken at baseline, rather than the later measures of stiffness.

**What do these findings mean?:**

- Faster stiffening of the aorta in mid-to-older ages may influence brain health, specifically, the brain’s delicate blood vessels and the connectivity between different brain regions.
- However, in order to benefit memory in older age, strategies to prevent or slow down arterial stiffening may be required even earlier in the life span.
- Although this was a large study, it was conducted in a relatively well-educated and largely male sample, so future research on more representative samples are needed to allow us to generalise the findings to the wider population.

## Introduction

Nearly 50 million people currently live with dementia worldwide, and this figure is expected to triple by 2050 [1]. While the exact mechanisms underlying dementia are still poorly understood, accumulating evidence implicates a number of potentially modifiable risk factors linked to another global health burden, cardiovascular disease (CVD) [2]. Timely prevention strategies targeting risk factors common to these diseases may therefore have a dual benefit in reducing 2 of the world’s most prevalent causes of morbidity and mortality.

One established risk factor for CVD is disproportionate stiffening of the aorta [3]. Aortic stiffness refers to the loss of elasticity in the artery wall and occurs gradually with age [4]. Acceleration of this process can also compromise the artery’s ability to dampen the pulsatile energy flowing from the heart to delicate target organs like the brain. Indeed, numerous studies have now demonstrated a relationship between aortic stiffness and accelerated cognitive decline, suggesting that this may also be an important risk factor for dementia ([5–8]; for review, see[9]). Less well understood, however, is the relationship of aortic stiffness with cerebrovascular and microstructural changes in the brain that may contribute to cognitive impairment and dementia [10].

Some magnetic resonance imaging (MRI) studies have revealed a link between higher aortic stiffness and markers of vascular pathology in the brain such as white matter lesions (WMLs), lacunar infarcts, and cerebral microbleeds ([11–13]; for review, see[14]). However, most of these studies have visually dichotomized brain tissue into normal and abnormal, which lacks quantitative and spatial detail about the specific brain regions that may be most sensitive to damage. Very few studies have examined brain microstructural [15,16] or cortical perfusion [17] correlates of aortic stiffness at a voxel level.

Moreover, to our knowledge, no human study to date has investigated the potential impact that faster rates of arterial stiffening have on these measures. An increased rate of arterial stiffening is commonly observed in the transition from mid-to-late life; a time when a combination of ageing and long-term CVD risk exposure interact to negatively influence distensibility of major arteries. Recent work in animal models has strengthened claims that this link between excess pulsatility and cerebral outcomes may be causal [18]. De Montgolfier and colleagues showed that surgically induced rapid increases in pulsatile pressure in mice results in endothelial dysfunction and hypoperfusion within the brain’s fragile microcirculation, all accompanied by significant declines in cognitive function [18].

Using repeat measures of carotid–femoral pulse wave velocity (PWV) measured as part of the long-running Whitehall II study, we assessed changes in arterial stiffness over 4 years and investigated their relationship with multimodal brain MRI and cognitive outcomes in a subset of 542 participants of the Whitehall II Imaging Sub-study.

## Methods

### Sample selection

Participants were drawn from the Whitehall II Study, a cohort of 10,308 British civil servants followed since 1985 [19]. Arterial stiffness was measured using carotid–femoral PWV at Phase 9 (2007 to 2009, *n* = 4,347) and again at Phase 11 (2012 to 2013, *n* = 4,344), and a total of 3,454 participants had repeat measures of PWV in the Whitehall II cohort [20]. This cohort has had a consistently high retention rate since its inception, with 87% of Phase 9 participants returning for a clinical visit at Phase 11. Following Phase 11, 774 randomly selected participants received multimodal brain MRI scans and cognitive tests at the Wellcome Centre for Integrative Neuroimaging, University of Oxford, as part of the Whitehall II Imaging Sub-study (2012 to 2016, protocol described previously[21]). Due to a scanner upgrade two-thirds of the way through the study, 2 MRI scanners were used: a 3T Siemens Magnetom Verio scanner (Erlangen, Germany) with a 32-channel head coil (*n* = 552, April 2012 to December 2014) and a 3T Siemens Prisma Scanner (Erlangen, Germany) with a 64-channel head–neck coil in the same centre (*n* = 222, July 2015 to December 2016) [21]. The scan parameters were identical or closely matched between scanners (S1 Text), and a scanner covariate was used in all analyses. Pseudocontinuous arterial spin labelling (pCASL) scans to quantify cerebral blood flow (CBF) were acquired in a subset of 145 participants who were all scanned on the Verio scanner.

Of the 774 participants, we excluded those with incidental findings on the MRI scan (e.g., large strokes, brain tumours, or cysts; *n* = 31), incomplete or poor quality T1 or diffusion tensor imaging (DTI) data (*n* = 59), incomplete PWV measurements at either Phase 9 or 11 (*n* = 140), and extreme outlying values of PWV at Phases 9 and 11, >4 SDs from the mean (*n* = 2). Accordingly, to maximise power for the voxel-wise cross-subject statistics, the volumetric and diffusion analyses were performed on 542 participants, while the WML analysis was performed in a subset of *n* = 533 with available FLAIR scans, cognitive analyses in a subset of *n* = 537 with all available cognitive data, and the perfusion analysis in a subset of *n* = 112 with available pCASL scans. None of the participants had dementia at the time of the MRI scan. A flowchart of the sample selection is in S1 Fig, and a comparison of the selected sample and full imaging cohort is presented in S1 Table. This study was not preregistered, but the hypothesis and analysis protocols are within the scope of planned hypotheses for this cohort, as published in the Whitehall II Imaging Study Protocol [21].

**Fig 1:**
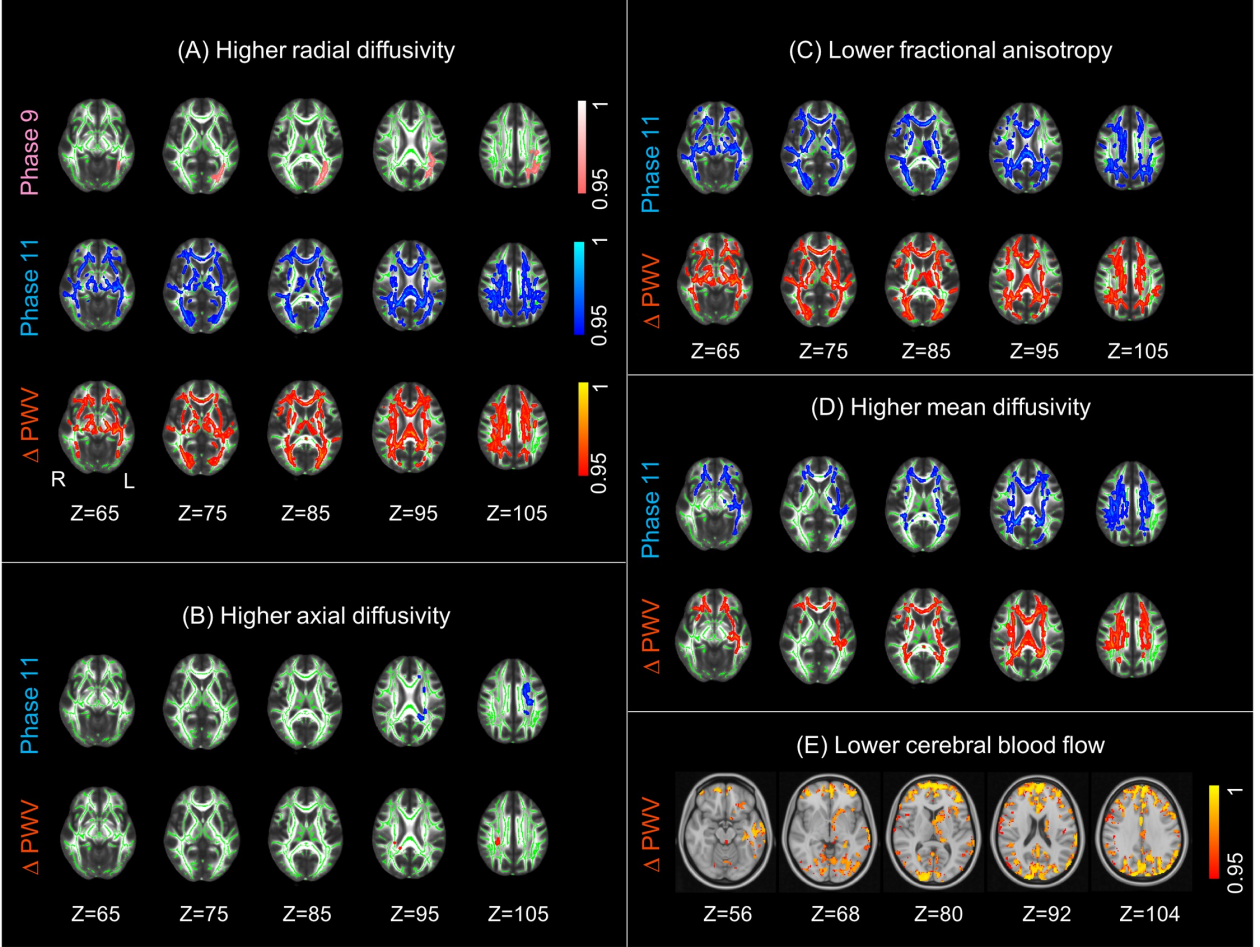
Association of aortic stiffening with WM microstructure and CBF. Higher aortic stiffening was associated with (A) higher RD, (B) higher AD, (C) lower FA, (D) higher MD, and (E) lower CBF. Associations with Phase 9 PWV, Phase 11 PWV, and ΔPWV are presented in pink, blue, and red yellow, respectively. Five horizontal slices are displayed with MNI152 coordinates ranging from Z = 65 to Z = 105 for WM and Z = 56 to Z = 104 for CBF. The WM clusters are overlaid on the study-specific mean FA skeleton (green) and the standard FMRIB58 FA image, and the CBF clusters are overlaid on the standard MNI152 brain. All results are thresholded at *p* < 0.05 (TFCE and FWE-corrected *p*-values) and are presented with a colour gradient for 1 *p*-values. AD, axial diffusivity; CBF, cerebral blood flow; FA, fractional anisotropy; FWE, family-wise error; L, left; MD, mean diffusivity; PWV, pulse wave velocity; R, right; RD, radial diffusivity; TFCE, threshold-free cluster enhancement; WM, white matter.

**Fig 2:**
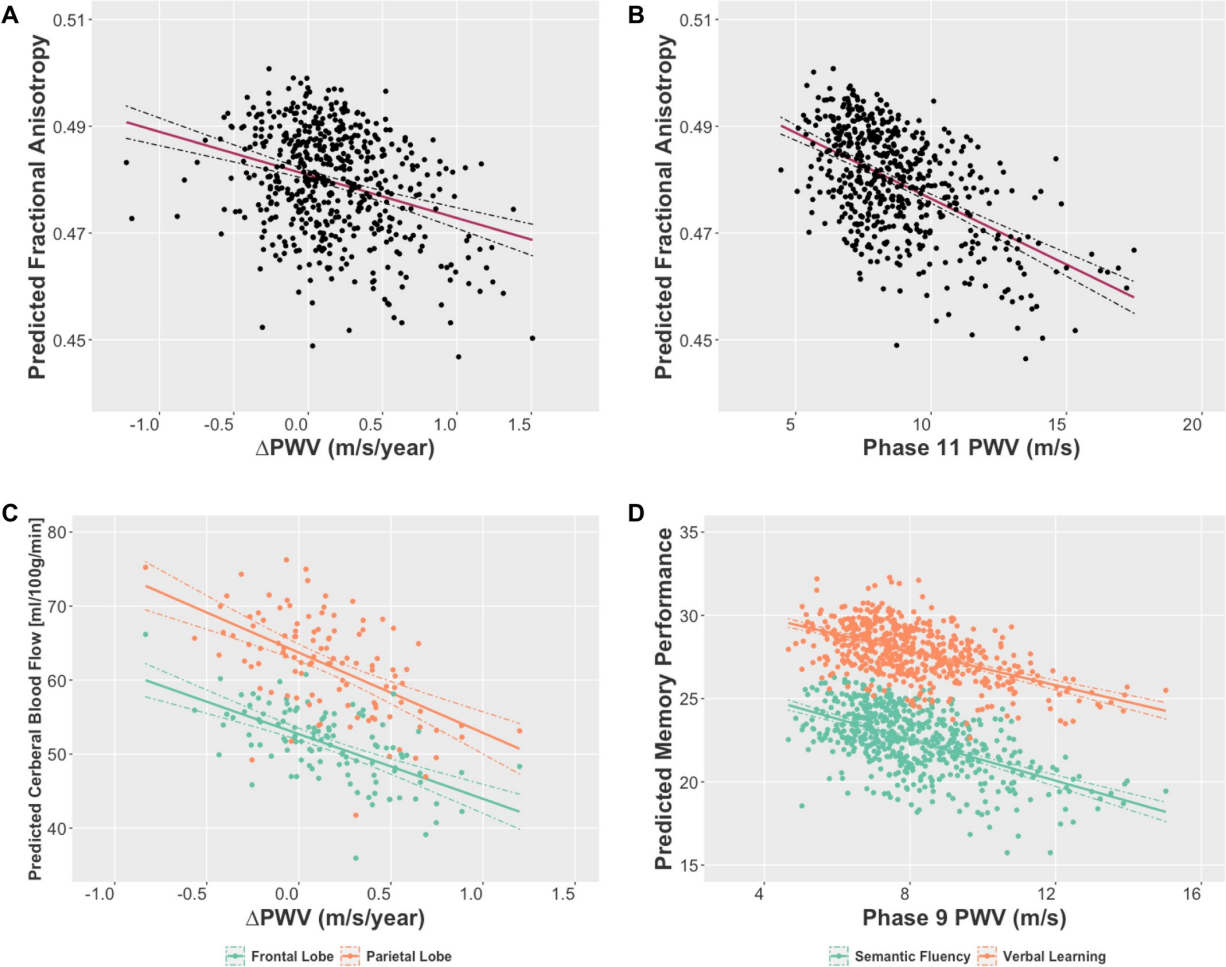
Partial regression plots showing the associations of PWV with brain and cognitive outcomes which survived Bonferroni correction for multiple comparisons. Plots present estimated marginal means for (A, B) Predicted global FA, (C) Predicted CBF from frontal (green) and parietal (orange) lobes, and (D) Predicted memory performance on semantic fluency (green) and verbal learning (orange) tests, plotted against (A,C) Rate of change of PWV, (B) PWV at Phase 11, and (D) PWV at Phase 9. Estimated marginal means are derived from linear regression models adjusted for age, MAP, BMI, and antihypertensive treatment measured at the respective phase (9 or 11), number of years from the respective phase (9 or 11) to the MRI scan, and sex, education, socioeconomic status, and MRI scanner model. BMI, body mass index; CBF, cerebral blood flow; FA, fractional anisotropy; MAP, mean arterial pressure; MRI, magnetic resonance imaging; PWV, pulse wave velocity.

Data analysis of the vascular, cognitive, and MRI data for this study was conducted between May 2018 and January 2020. The manuscript has been modified from its original submission in accordance with peer review in August 2020, with the addition of new supplementary files (S1–S3 Tables and S2 Text) and the revision of Tables 1–3, Figs 1 and 2, and S1 and S2 Figs as a result of the exclusion of 2 participants who had outlying values of PWV at both phases. Outlier removal strengthened all the associations reported in the original manuscript but did not alter the overall conclusions. This study is reported as per the Strengthening the Reporting of Observational Studies in Epidemiology (STROBE) guideline for cohort studies.

**Table 1:** Characteristics of the 542 participants at Phase 9 and Phase 11.

Sample characteristics	Phase 9 2007–2009	Phase 11 2012–2013
*N*	542	542
Age, years	63.9 (5.2)	68.0 (5.2)
Age range, years	55.6–77.4	59.8–81.8
*N* male (%)	444/542 (81.9%)	
PWV m/s	8.1 (1.7)	8.7 (2.1)
PWV range m/s	4.7–15.0	4.5–17.5
Mean change in PWV between Phase 9 and Phase 11, m/s/year	0.2 (0.4)	
Systolic blood pressure, mmHg	122.5 (14.7)	125.1 (15.4)
Diastolic blood pressure, mmHg	70.6 (10.1)	70.3 (9.5)
MAP, mmHg	87.9 (11.0)	88.6 (10.7)
BMI, kg/m^2^	25.9 (3.6)	25.9 (3.6)
Antihypertensive treatment, *N* (%)	137/542 (25.3%)	175/542 (32.3%)
Current smokers, *N* (%)	29/538 (5.4%)*	20/541 (3.7%)*
Type 2 diabetes, *N* (%)	8/542 (1.5%)	37/539 (6.9%)*
Current CVD (angina, stroke, or myocardial infarction), *N* (%)	12/542 (2.2%)	16/542 (3.0%)

Values represent mean (SD) or no. (%).

* indicates the presence of missing data.

BMI, body mass index; CVD, cardiovascular disease; MAP, mean arterial pressure; *N*, number; PWV, pulse wave velocity; SD, standard deviation.

**Table 2:** Summary of the brain and cognitive outcome variables examined at the MRI phase (2012–2016).

OVERVIEW (*N* = 542)	Mean (SD)
Age at MRI phase, years	69.79 (5.22)
Years of education	14.24 (3.01)
Years from Phase 9 to MRI phase	5.90 (1.40)
Years from Phase 11 to MRI phase	1.82 (1.36)
Years from Phase 9 to Phase 11	4.08 (0.18)
**STRUCTURAL OUTCOMES (*N* = 542)**	
GM volume, % ICV	51.5 (4.2)
Global FA	0.48 (0.02)
Global MD (× 10^−3^)	0.69 (0.03)
Global RD (× 10^−3^)	0.49 (0.03)
Global AD (× 10^−3^)	1.08 (0.03)
WMLs, % ICV^**a**^	0.53 (0.33)
**CBF OUTCOMES (CBF, *N* = 112)**	
Frontal lobe CBF (ml/100 g/min)	51.37 (12.17)
Parietal lobe CBF (ml/100 g/min)	62.06 (15.49)
Occipital lobe CBF (ml/100 g/min)	49.04 (18.82)
Temporal lobe CBF (ml/100 g/min)	49.35 (10.17)
**COGNITIVE OUTCOMES (*N* = 537)**	
Verbal learning (no. of words recalled, short term)	27.81 (4.37)
Delayed verbal recall (no. of words recalled, delayed)	9.45 (2.51)
Semantic fluency (no. of animals listed)	22.51 (5.28)
Short-term memory (total digit span score)	31.08 (5.47)
Executive function (Trail Making Test B-A, min)	0.58 (0.44)
Executive function (digit substitution score)	63.45 (13.05)
MoCA (total score)	27.39 (2.10)

^a^Analysed WML subset (*N* = 533).

Values represent mean (SD). GM volume and WMLs are expressed as % of total intracranial volume.

AD, axial diffusivity; CBF, cerebral blood flow; FA, fractional anisotropy; GM, grey matter; ICV, intracranial volume; MD, mean diffusivity; MoCA, Montreal Cognitive Assessment; MRI, magnetic resonance imaging; RD, radial diffusivity; SD, standard deviation; WML, white matter lesion.

**Table 3:** Associations between aortic PWV and global brain and cognitive outcomes.

	Model 1: Phase 9 PWV (m/s)		Model 2: ΔPWV (m/s/y)		Model 3: Phase 11 PWV (m/s)	
	B [95% CI]	*p*	B [95% CI]	*p*	B [95% CI]	*p*
**1. Brain structure (*N* = 542)**						
**FA [**× 10^3^]	−0.90 [−1.90, 0.09]	0.08	−5.65 [−9.75, −1.54]	0.007^*^	−1.12 [−1.95, −0.29]	0.009^*^
**MD** [× 10^6^]	1.19 [−0.19, 2.57]	0.09	5.66 [−0.06, 11.38]	0.05	1.22 [0.06, 2.38]	**0.04**
**RD** [× 10^6^]	1.39 [−0.10, 2.88]	0.07	7.50 [1.36, 13.64]	**0.017**	1.56 [0.32, 2.81]	**0.014**
**AD** [× 10^6^]	0.79 [−0.54, 2.12]	0.25	1.97 [−3.56, 7.50]	0.48	0.54 [−0.59, 1.66]	0.35
**GM (%ICV)**	−0.06 [−0.27, 0.16]	0.60	−0.41 [−1.30, 0.48]	0.37	−0.09 [−0.27, 0.09]	0.32
**WML (%ICV, *N* = 533)**	0.001 [−0.02, 0.02]	0.91	0.03 [−0.04, 0.10]	0.39	0.002 [−0.01, 0.02]	0.78
**2. CBF (ml/100 g/min, *N* = 112)**						
**Frontal lobe**	0.59 [−1.14, 2.32]	0.50	−10.85 [−17.91, −3.79]	0.003^*^	−1.05 [−2.43, 0.33]	0.14
**Temporal lobe**	0.61 [−0.80, 2.01]	0.39	−7.14 [−12.94, −1.35]	**0.016**	−0.42 [−1.55, 0.72]	0.47
**Parietal lobe**	0.50 [−1.65, 2.66]	0.65	−12.75 [−21.58, −3.91]	0.005^*^	−1.28 [−3.00, 0.45]	0.15
**Occipital lobe**	0.61 [−1.94, 3.17]	0.64	−13.30 [−23.87, −2.73]	**0.014**	−1.41 [−3.43, 0.62]	0.17
**3. Cognitive performance (*N* = 537)**						
**Semantic fluency**	−0.47 [−0.76, −0.18]	0.001^*^	0.89 [−0.32, 2.11]	0.15	−0.12 [−0.37, 0.12]	0.32
**Verbal learning**	−0.36 [−0.60, −0.12]	0.003^*^	0.44 [−0.55, 1.43]	0.38	−0.10 [−0.30, 0.10]	0.33
**Delayed verbal recall**	−0.11 [−0.25, 0.03]	0.11	0.18 [−0.40, 0.77]	0.54	−0.01 [−0.13, 0.11]	0.91
**Short-term memory (digit span)**	−0.03 [−0.33, 0.28]	0.86	−0.19 [−1.46, 1.08]	0.77	−0.08 [−0.34, 0.17]	0.52
**Executive function (trail making)**	−0.001 [−0.03, 0.02]	0.91	−0.01 [−1.11, 0.09]	0.84	−0.002 [−0.02, 0.02]	0.85
**Executive function (digit substitution)**	−0.33 [−1.04, 0.39]	0.37	1.54 [−1.42, 4.50]	0.31	−0.05 [−0.66, 0.55]	0.86
**Global cognition (MoCA)**	−0.12 [−0.24, −0.01]	**0.04**	−0.08 [−0.57, 0.41]	0.75	−0.07 [−0.17, 0.03]	0.17

Models 1 and 3 were adjusted for age, MAP, BMI, and antihypertensive use at the respective phase (9 or 11), years from the respective phase to the MRI scan and scanner model, sex, education, and socioeconomic grade. Model 2 was adjusted for the aforementioned covariates as well as Phase 9 PWV and years from P11 to MRI to examine the unique contribution of rate of stiffening (regardless of baseline). Unstandardised B coefficients (B) and 95% CIs are reported, representing mean change in outcome per 1 m/s increase in PWV (for Models 1 and 3) and per 1m/s/year increase in rate of change of PWV (for Model 2). GM volume and WMLs are expressed as percentages of ICV; CBF is expressed in ml/100 g tissue/min; PWV in m/s, ΔPWV in m/s/year. FA values were multiplied by 103 and RD, MD, AD values by 106.

* indicates associations which survive post hoc Bonferroni corrections for multiple comparisons.

AD, axial diffusivity; BMI, body mass index; CBF, cerebral blood flow; CI, confidence interval; FA, fractional anisotropy; GM, grey matter; ICV, intracranial volume; MAP, mean arterial pressure; MD, mean diffusivity; MoCA, Montreal Cognitive Assessment; MRI, magnetic resonance imaging; PWV, pulse wave velocity; RD, radial diffusivity; WML, white matter lesions.

### Ethics statement

The study was approved by the University of Oxford Medical Sciences Interdivisional Research Ethics Committee (Reference: MS IDREC-C1-2011-71) and the University College London Committee on the Ethics of Human Research (Reference: 85/0938). Written informed consent was obtained from all participants.

### Pulse wave velocity

The protocol for measuring PWV in the Whitehall II Study has been described previously [6]. Briefly, aortic PWV was assessed between carotid and femoral sites using applanation tonometry (SphygmoCor; Atcor Medical, Australia). At each phase, 2 measurements of PWV were acquired from each participant, and a third measurement was taken if the difference between the first 2 measurements was >0.5 m/s. Path length was determined with a tape measure by subtracting the carotid–sternal notch distance from the femoral–sternal notch distance. Repeat measures of PWV at Phases 9 and 11 yielded a bias and 95% limits of agreement of 0.03 (−2.3, 2.4) and −0.01 (−2.6, 2.6) m/s, respectively. The average of the measurements at each phase was used in cross-sectional analyses. The change (ΔPWV) was calculated as the difference between phases divided by time between visits (m/s/year), with positive values representing faster rates of stiffening.

### Neuroimaging outcomes

Detailed protocols for the acquisition and preprocessing of all scans have been described previously[21] and in S1 Text. All images were processed using FMRIB Software Library (FSL) tools. Briefly, grey matter (GM) volumes were assessed using voxel-based morphometry (FSL-VBM) analyses on structural T1-weighted scans [22]. WMLs were quantified as hyperintensities on fluid-attenuated inversion recovery (FLAIR) scans using the FSL-BIANCA tool [23]. CBF was quantified as the rate of delivery of arterial blood to brain tissue (ml of blood per 100 g of tissue per minute) using pCASL scans analysed with the FSL-BASIL toolkit [24]. White matter (WM) microstructure was assessed using DTI scans analysed with tract-based spatial statistics (FSL-TBSS) [25]. DTI is sensitive to directional diffusion of water within the axon, which is unrestricted along the axon, but hindered perpendicularly due to the presence of the myelin sheath. Directionality and amount of water diffusion is quantified by DTI-derived metrics such as fractional anisotropy (FA), radial diffusivity (RD), axial diffusivity (AD), and mean diffusivity (MD).

Decreases in FA alongside increases in diffusivity are established and sensitive measures of WM damage in dementia [26]. Lower CBF and higher WMLs are also well-established surrogate neuroimaging end points for dementia [27].

In addition to voxel-based analyses, we also examined summary measures of global brain physiology. For each participant, total GM and WML volumes were extracted using FSL-FAST and FSL-BIANCA, respectively. These were normalised to total intracranial volume and expressed as GM% and WML%. Global FA, MD, RD, and AD were extracted from the mean TBSS skeleton, with values ranging from 0 (representing isotropic diffusion) to 1 (representing anisotropic diffusion). Total CBF from the frontal, temporal, parietal, and occipital lobes were extracted from native-space CBF maps using MNI152 masks and expressed in ml/100 g/min.

### Cognitive outcomes

We examined performance on 7 cognitive domains: semantic fluency: number of animals named in 1 minute; verbal learning: number of words learned after 3 trials of the Hopkins Verbal Learning Test–Revised (HVLT-R); delayed verbal recall: number of words recalled after a 30-minute delay on HVLT-R; working memory: total score on the Digit Span Forward, Backward, and Sequence tests; executive function: difference in time to completion between Trail Making Test B and A and digit substitution test (Wechsler Adult Intelligence Scale-Fourth Edition (WAIS-IV)); and global cognitive function: Montreal Cognitive Assessment (MoCA).

### Statistical analysis

All exposures and outcomes were treated as continuous measurements. Longitudinal change in PWV between Phase 9 and Phase 11 was analysed using an ANCOVA covarying for baseline age and sex.

Cross-subject voxel-wise regressions were performed separately on spatial maps of GM volume, WM microstructure (FA, MD, RD, and AD) and CBF (preprocessing detailed in S1 Text). For each modality, the respective spatial maps for all participants were concatenated into a 4D file. This was submitted to the FSL-Randomise tool, which is a nonparametric permutation-based method used for inference (thresholding) on statistical maps when the null distribution is not known (https://fsl.fmrib.ox.ac.uk/fsl/fslwiki/Randomise/Theory, [28]). It allows the use of a standard general linear model (GLM) design matrix to model explanatory variables (in this case PWV and covariates). Here, we used 5,000 permutations in FSL-Randomise, which greatly reduces the margin of error for the nominal alpha, and examined the threshold-free cluster enhancement (TFCE)-based *t* tests, which are more sensitive and interpretable than cluster-based thresholding [29]. Three GLMs were run to assess the associations of Phase 9 PWV (Model 1), ΔPWV (Model 2), and Phase 11 PWV (Model 3) on the aforementioned MRI and cognitive outcomes. All models included the covariates listed below. For the MRI outcomes, voxel-wise results are displayed at a TFCE-corrected and family-wise error (FWE)-corrected significance threshold of *p* < 0.05, corrected for multiple voxel-wise comparisons. This represents a 95% confidence of no false positives in the significant clusters identified.

Statistical analyses of MRI-derived variables were performed in R (Version 3.5.2). Four CBF regions of interest (frontal, temporal, parietal, and occipital lobes), 4 global WM metrics (global FA, MD, RD, and AD), and 7 cognitive tests were used as dependent variables in separate multivariate GLMs to test for associations with Phase 9 PWV (Model 1), ΔPWV (Model 2), and Phase 11 (Model 3). In each case, if the multivariate model was significant, post hoc univariate regressions were interpreted to assess contributions of the individual CBF, WM, and cognitive measures. Post hoc tests were considered statistically significant at a strict Bonferroni-corrected threshold of *p* < 0.05/4 (i.e., *p* < 0.0125) to correct for multiple comparisons across the 4 WM and CBF metrics and *p* < 0.05/7 (i.e., *p* < 0.007) for the 7 cognitive tests. Statistical summaries for results which did not survive Bonferroni correction are reported for completion. Additionally, the associations of Phase 9 (Model 1), ΔPWV (Model 2), and Phase 11 PWV (Model 3) with WML% and GM% were assessed in separate univariate linear regression models. All models included the covariates listed below.

We further assessed whether associations of PWV with cognition were mediated by brain MRI markers using a mediation analysis in PROCESSv3.4 for SPSS (2012 to 2019 by Andrew F. Hayes; detailed in S2 Text).

### Covariates

Covariates were selected a priori based on the literature. All models (presented in Table 3) included sex, total years of full-time education (self-reported at the MRI phase), socioeconomic status (defined based on the highest civil service employment grade achieved at Phase 3 in 2002 to 2004; 1 = highest grade; 2 = intermediate, and 3 = lowest grade), and MRI scanner model, as well as the following covariates measured at Phase 9 (for Models 1 and 2) or Phase 11 (for Model 3): age, mean arterial pressure (MAP), body mass index (BMI), current antihypertensive treatment (self-report), and number of years from the respective phase (9 or 11) to the MRI scan. Model 2 (ΔPWV) also covaried for Phase 9 PWV, in order to assess the brain/cognitive correlates of rate of aortic stiffening regardless of baseline measures. In order to confirm that observed associations with ΔPWV (i.e., Model 2) were not driven by the inclusion of baseline PWV in the model, we also tested 2 additional models, all of which produced equivalent results (S3 Table): First, we tested associations of ΔPWV without including baseline PWV as a covariate, and secondly, we used the residuals from the regression of Phase 11 PWV on Phase 9 PWV in the place of ΔPWV—a technique which has the advantage of using an exposure which lies orthogonal to, and is therefore unrelated to, baseline values (S3 Table). We note that all models produced equivalent results, thus ensuring the robustness of our findings. Additional covariates such as self-reported current smoking status (yes/no), self-reported current CVD (defined as having been diagnosed with myocardial infarction, angina, or stroke at a clinical examination), self-reported history of type 2 diabetes (yes/no) were tested in a stepwise forward linear regression and as they did not significantly improve model fit they were not included in the final results described in Table 3 in order to avoid overfitting.

## Results

### Participant characteristics

Participant characteristics are in Tables 1 and 2; 81.9% of the participants were male, and 58.9%, 38.2%, and 3.0% of participants were categorised in socioeconomic grades 1, 2, and 3, respectively. There was a significant increase in PWV during the 4-year follow-up from Phase 9 to 11 with a mean increase of 0.7 m/s (SD = 1.6, *p* < 0.0001) between phases, after correcting for baseline age and sex. On average, PWV increased by 0.2 (SD = 0.4) m/s per year (S2 Fig).

The samples in the structural (*n* = 542) and perfusion MRI analysis (*n* = 112) were representative of the parent Whitehall Imaging cohort (*n* = 774) in key characteristics such as age, years of education, MAP, current antihypertensive treatment, proportion of smokers, and those with current CVDs (S1 Table). However, the selected samples had a smaller proportion of females and a lower BMI than the parent cohort. We note that the absolute differences in these measures were very small (approximately 4% fewer females and 0.5 kg/m^2^ reduction in BMI in the structural sample compared to the parent cohort); nonetheless, BMI and sex have been included as covariates in all analyses of PWV with brain and cognitive outcomes.

### Association of PWV with brain microstructure

Higher ΔPWV (p_(model)_ = 0.003) and higher Phase 11 PWV (p_(model)_ = 0.004) predicted poor overall WM microstructure in covariate-adjusted multivariate GLMs with global FA, MD, RD, and AD as dependent variables. Post hoc univariate regressions revealed that this was driven by associations of aortic stiffening with lower global FA (B [95% CI] for ΔPWV: −5.65 [−9.75, −1.54] and Phase 11 PWV: −1.12 [−1.95, −0.29]), higher global MD (B [95% CI] for ΔPWV: 5.66 [−0.06, 11.38] and Phase 11 PWV: 1.22 [0.06, 2.38]), and higher global RD (B [95% CI] for ΔPWV: 7.50 [1.36, 13.64] and Phase 11 PWV: 1.56 [0.32, 2.81]); however, only the associations with global FA survived Bonferroni correction for multiple comparisons (*p* < 0.0125, Table 3 and Fig 2).

Voxel-wise analyses demonstrated that aforementioned associations of ΔPWV with FA, MD, and RD were localised to 23.9%, 11.8%, and 22.2% of WM tracts, respectively, covering the corpus callosum, fornix, anterior thalamic radiation, superior and posteriori corona radiata, superior longitudinal fasciculus, and corticospinal tracts (Table 4). Similar widespread associations were observed for Phase 11 PWV, covering 22.3%, 13.6%, and 24.1% of WM for FA, MD, and RD, respectively (Table 4). For both ΔPWV and Phase 11 PWV, voxel-wise analyses revealed additional, localised, and less pronounced associations with AD within the uncinate fasciculus and posterior corona radiata (Fig 1 and Table 4). Furthermore, while evidence of associations were also seen with baseline Phase 9 PWV, these were less pronounced both with the extracted WM measures [Phase 9 PWV: (p_(model)_ = 0.18), Table 3] and voxel-based analyses (Fig 2, covering only 1.4% of WM tracts for RD).

**Table 4:** Cluster report for the voxel-wise associations of PWV with FA, MD, RD, AD, and CBF.

Outcome	Cluster no.	Number of voxels (% of WM tract)	*p*-value	Coordinates of maxima (X, Y, Z)	Location of maxima
**1. Association with ΔPWV**					
FA	1	37,544/159,400 (23.6%)	0.004	73, 103, 105	Body of corpus callosum
FA	2	45/159,400 (0.03%)	0.049	75, 83, 40	Right corticospinal tract (middle cerebellar peduncle)
MD	1	18,853/159,400 (11.8%)	0.019	66, 95, 100	Right posterior corona radiata
RD	1	35,406/159,400 (22.2%)	0.01	73, 106, 105	Body of corpus callosum
AD	1	144/159,400 (0.09%)	0.046	67, 97, 101	Right posterior corona radiata
CBF	1	37,835/206,802 (18.3%)	0.002	46, 95, 41	Frontal pole
CBF	2	127/206,802 (0.06%)	0.031	18, 61, 25	Right middle temporal gyrus
CBF	3	34/206,802 (0.02%)	0.044	20, 51, 51	Right parietal operculum
**2. Association with Phase 11 PWV**					
FA	1	34,854/159,400 (21.9%)	0.01	123, 63, 73	Left posterior thalamic radiation
FA	2	683/159,400 (0.4%)	0.047	100, 96, 136	Corticospinal tract
MD	1	21,745/159,400 (13.6%)	0.016	115, 115, 102	Left superior corona radiata
RD	1	38,470/159,400 (24.1%)	0.01	125, 124, 54	Left uncinate fasciculus
AD	1	269/159,400 (0.2%)	0.046	110, 125, 105	Left superior corona radiata
AD	2	209/159,400 (0.1%)	0.046	113, 89, 103	Left posterior corona radiata
AD	3	177/159,400 (0.1%)	0.045	115, 133, 105	Left superior corona radiata
**3. Association with Phase 9 PWV**					
RD	1	1,962/159,400 (1.2%)	0.041	123, 71, 99	Left superior longitudinal fasciculus
RD	2	272/159,400 (0.2%)	0.049	114, 93, 123	Corticospinal tract

A total of 159,400 and 206,802 voxels were investigated for the WM tracts and CBF analyses, respectively. Cluster sizes (number of significant voxels and % of WM or GM area covered), TFCE and FWE-corrected *p*-values, voxel coordinates in MNI152 space, and locations of cluster maxima are reported for all significant clusters.

AD, axial diffusivity; CBF, cerebral blood flow; FA, fractional anisotropy; FWE, family-wise error; GM, grey matter; MD, mean diffusivity; PWV, pulse wave velocity; RD, radial diffusivity; TFCE, threshold-free cluster enhancement; WM, white matter.

Neither cross-sectional nor longitudinal aortic PWV were associated with WML%, total GM% (Table 3), or GM volume assessed with voxel-wise analysis.

### Association of PWV with cerebral perfusion

Higher ΔPWV was associated with lower total CBF in the frontal (B [95% CI]: −10.85 [−17.91, −3.79]), temporal (B [95% CI]: −7.14 [−12.94, −1.35]), parietal (B [95% CI]: −12.75 [−21.58, −3.91]), and occipital lobes (B [95% CI]: −13.30 [−23.87, −2.73]) in the subset of 112 participants with perfusion imaging scans; however, only associations with frontal and parietal lobe CBF survived Bonferroni correction for multiple comparisons (*p* < 0.0125, Table 3 and Fig 2). The voxel-wise analyses revealed most pronounced effects in the parietal, lateral occipital, and cuneal cortex, occipital pole, right middle temporal gyrus, frontal pole, and the precuneus (Fig 1). Cross-sectional PWV at Phases 9 and 11 were not significantly associated with regional CBF in the voxel-wise analysis or using extracted mean lobar CBF (Table 3).

### Association of PWV with cognitive performance

Baseline PWV at Phase 9 was associated with lower overall cognitive performance at the MRI phase (p_(model)_ = 0.01) in covariate-adjusted multivariate GLMs. Post hoc linear regressions revealed that this association was driven by poorer performance on the semantic fluency and verbal learning tests, which survived a Bonferroni-corrected threshold for significance (*p* < 0.007, Table 3 and Fig 2). There was also some evidence for an association with lower MoCA scores; however, this did not survive Bonferroni corrections (*p* < 0.05, Table 3). In contrast, neither Phase 11 PWV (p_(model)_ = 0.71) nor ΔPWV (p_(model)_ = 0.76) were associated with cognitive performance at the MRI visit, and while the post hoc univariate regressions are presented for completion, these are not further interpreted (Table 3). Moreover, the associations of Phase 9 PWV with cognitive outcomes were not mediated by WM microstructure (S2 Table).

## Discussion

In this study, we show that an increased rate of arterial stiffening is associated with lower WM microstructural integrity and CBF in older age. Furthermore, these associations were present in diffuse brain areas, suggesting that exposure to excess pulsatility may result in a widespread damaging effect on the fragile cerebral microstructure. Cognitive function at follow-up related more closely with baseline arterial stiffness rather than rate of arterial stiffening. Taken together, these findings suggest that although faster rates of arterial stiffening in the transition to old age may negatively impact brain structure and function, long-term exposure to higher levels of arterial stiffness prior to this point may be the most important determinant for future cognitive ability.

While aortic stiffening has predominantly been studied in the context of CVD, recent evidence suggests that large artery dysfunction may also play a role in dementia [30]. Indeed, patients with Alzheimer’s disease and vascular dementia reportedly have higher levels of aortic stiffness relative to cognitively healthy adults [30]. Aortic stiffening is a hallmark of vascular ageing and may lead to a heightened state of oxidative and inflammatory damage within the cerebral tissues due to an increased penetrance of excess pulsatility into the fragile microcirculation of the brain.[9] These changes have been shown to disrupt endothelial cell function and the blood brain barrier in animal models and have also been hypothesised to compromise cerebral perfusion and ultimately lead to amyloid deposition, neurodegeneration, and cognitive impairment.[9,31] While previous studies have related cross-sectional measures of arterial stiffness to cognition, this is the first study, to our knowledge, to publish associations between progressive increases in aortic stiffening over a 4-year period and cerebral and cognitive outcomes in later life.

We report a number of novel findings. First, faster rates of arterial stiffening from average age 64 to 68 years, and higher follow-up aortic stiffness at approximately 68 years were associated with widespread WM microstructural decline within the corpus callosum, corona radiata, superior longitudinal fasciculus, cortico–spinal tracts, and internal capsule. Observations of lower FA with a concomitantly higher diffusivity within these tracts are suggestive of axonal degeneration and loss of myelin. Notably, these tracts have also been shown to be vulnerable to arterial stiffness in 2 smaller studies in older adults [15,16]. These tracts are supplied by the anterior and middle cerebral arteries and given that they are also compromised in Alzheimer’s and vascular dementia [26], they may be potential markers of the role of arterial stiffening in dementia pathophysiology. However, in our study, associations of arterial stiffness at the younger baseline age of 64 with WM were weaker, more localised, and only restricted to voxel-wise increases in RD compared to the more widespread WM impairments linked to the later measurements of PWV. This is somewhat in contrast to findings from the Framingham Heart Study, which report associations of PWV with lower FA in the corpus callosum and corona radiata even in younger adults (approximately 46 years old) [16]. They also note, however, that their observed associations with FA were accentuated by age. Cohort biases may play a role in reconciling these inconsistencies; however, it is also plausible that while regional (and perhaps subthreshold) PWV-related changes in cerebral microstructure can begin in early-to-mid life, these changes become more widespread and pronounced as rates of aortic stiffening increase in the transition from mid-to-late life.

Second, in addition to evidence of disruptions to WM structural integrity, we also noted significantly lower CBF in posterior cingulate, parietal, medial temporal, frontal, and occipital cortices with faster rates of aortic stiffening in a subset of 112 participants. These results are in line with a recent cross-sectional study in individuals with mild cognitive impairment [17] and implicate brain areas typically affected by vascular ageing [32]. Similar to WM metrics, these effects were found to be 5 to 10 times larger for rates of stiffening than cross-sectional PWV, suggesting that exposure to rapid increases in pulsatile stress may be particularly damaging to cerebral perfusion compared with more gradual changes over time.

Third, we observed no associations of aortic stiffness with WMLs and GM density. Previous studies which have measured artery stiffness using brachial–ankle PWV or augmentation index have reported associations with WML load in community-dwelling adults [33] and in hypertensive populations [11]. Self-selection bias arising from patients volunteering to attend this imaging sub-study means that it is possible that our cohort—which compared to the general UK population was already relatively high functioning and with a healthier vascular profile [19]—had a relatively lower WML burden. Thus, changes in WM diffusion metrics (which have been noted to precede larger volumetric and pathological lesion changes) may have been more sensitive to the underlying structural tissue alterations at a voxel level than macroscopic WMLs. Moreover, previous voxel-based analyses of GM density have yielded mixed results; the Reykjavik Study reported no relationship between elevated aortic stiffness and GM in older adults [12], whereas the Framingham Heart Study revealed an association with lower thalamic volume in middle-aged adults [34]. Future studies examining longitudinal changes in GM atrophy or WML burden would be more informative than measures from a single time point.

Fourth, we observed an inverse association between baseline PWV and semantic fluency and verbal learning outcomes—alongside some evidence of an association with poor performance on the MoCA—a commonly used test for amnestic mild cognitive impairment [35]. These associations were not mediated by cerebral microstructure and are in agreement with numerous other studies linking cross-sectional measures of arterial stiffness in mid-life to later cognitive function [6], including studies in which similar relationships to verbal learning [7], episodic memory [8], and amnestic mild cognitive impairment have already been reported [36]. In contrast to our observed relationships between arterial stiffness and cerebral structure and perfusion, the cognitive outcomes were not related to later-life arterial stiffening but instead to baseline PWV alone. It is worth noting that previous reports from the Rotterdam Study and the Sydney Memory and Aging Study, which are closer in age to our Phase 11 sample (mean age approximately 68), have observed no independent associations of PWV with a similarly detailed cognitive battery examining attention, memory, and executive function in older adults [37,38], whereas the Framingham Heart Study of young adults (approximately 46 years) has reported associations with poorer processing speed and executive function [39]. Together with their findings, our results support the growing understanding that it is long-term cumulative exposure to both social (e.g., education and socioeconomic status) and physiological (inflammation, metabolic, vascular, and arterial stiffening) risk factors in the years preceding middle age—rather than the contemporary later-life changes—that are likely to represent the most important risk factor for overall cognitive ability in old age [1]. The fact that we observed considerably weaker associations between follow-up PWV and future cognitive outcomes suggests that earlier measures of PWV taken before the potential occurrence of rapid increases in arterial stiffness may represent a more effective biomarker for cognitive dysfunction risk stratification. Further studies aimed at untangling the cross-sectional and longitudinal associations of aortic stiffness with cognitive and cerebral phenotypes are required to confirm this.

Our findings suggest 2 things. First—and in agreement with previous studies linking modifiable risk factors to later adverse outcomes—early prevention strategies to reduce life-term exposure to risk factors may be required to in order to offer maximal benefits to later-life cognition, particularly in relation to domains such as semantic fluency and verbal learning. Second, novel to this study is our observation of additional relationships between faster rates of arterial stiffening during this period of life and the presence of pathological differences in WM structure and cerebral perfusion observed in the following years. These findings may provide the first evidence of the potential cerebral mechanisms underlying an accelerated rate of cognitive decline (as opposed to cross-sectional cognitive ability) during this time frame, which has previously been reported in individuals with faster rates of stiffening in the Whitehall II [40] and other cohorts [41]. We show for the first time that interventions to reduce or prevent the rapid increases in arterial pulsatility in mid-to-late life may reduce detrimental changes in WM integrity and blood flow, which have previously been linked to cognitive function, and may therefore also offer additional (albeit possibly more modest) benefits to cognitive ability in older age.

### Strengths and limitations

This is 1 of the largest MRI cohorts with multimodal brain imaging phenotyping, extensive cognitive testing, and longitudinal arterial stiffness measures. Moreover, arterial stiffness was measured 6 years prior to the MRI scan, starting at a mean age of 64 years when vascular risk has been demonstrated to contribute to dementia pathology. Importantly, we report associations of PWV over and above those of traditional vascular risk factors such as MAP, BMI, and antihypertensive use, suggesting that our results reflect true intrinsic effects of aortic stiffening, thus strengthening its role as a biomarker of brain and cognitive decline.

However, as with all observational cohort studies, it was not possible to infer causal associations between these exposures and outcomes. We were also unable to examine the role of the apolipoprotein E4 (APOE4) allele on our observed associations, given the substantial proportion of participants (nearly 25% of cohort) with missing APOE information. Future studies should consider investigating this allele given its established risk for CVD and dementia [42]. To account for the extensive cerebral phenotyping across measures of microstructure and perfusion, we performed strict corrections for multiple comparisons, and results that do not survive these corrections must therefore be interpreted with caution. Moreover, the generalizability of our findings may be limited by the fact that while the Whitehall II Study comprises community-dwelling adults, it might not be entirely representative of the UK population. First, the Whitehall II cohort is predominantly male (reflecting the makeup of the British Civil Service in 1985). It is well established in the literature that women show greater increases in arterial pulse pressure from midlife onwards compared to men and that this may occur through different underlying mechanisms to those captured using carotid–femoral PWV as employed here [43]. As such, care should be taken when generalising our findings to both sexes, and future reproduction studies in gender balanced cohorts will be needed to clarify our results. Second, given the relatively high educational attainment in this cohort, it possible that we have underestimated the observed association between PWV and cognitive outcomes. Third, while the sample analysed in this study was largely representative of the parent Whitehall Imaging cohort in key demographics and cardiovascular measures, it had a higher proportion of males and lower BMI than the original cohort. However, as we have adjusted for sex, BMI, and education in all our regression models, it is unlikely that these variables have significantly driven our results. Fourth, while we observed widespread and robust associations of aortic stiffening with cerebral hypoperfusion, we note that this analysis was only conducted in a small subset (20%) of the full sample. Thus, our findings must be replicated in independent studies before generalising to the wider population.

## Conclusions

To our knowledge, we have provided the first evidence of a relationship between faster rates of aortic stiffening during a 4-year period in mid-to-late life and detrimental changes to cerebral WM microstructural integrity and perfusion. These preliminary findings suggest that diffusion and perfusion MRI may be sensitive markers of recent damage caused by aortic stiffening and could therefore be potentially relevant outcome measures in intervention studies. However, as measures of cognition were found to most closely relate to baseline measures of stiffness, such interventions may need to be targeted earlier in life in order to offer maximal benefit for both vascular and cognitive health.

## Supporting information

S1 Fig Flowchart for sample selection. (TIF)

S2 Fig Distribution of the rate of change of aortic stiffening for 542 participants. Mean change was 0.2 ± 0.4 m/s per year, ranging from −1.2 to 1.5.
(TIF)

S1 Text MRI image acquisition and MRI image analysis. MRI, magnetic resonance imaging.
(DOCX)

S2 Text Mediation analysis. (DOCX)

S1 Table Sample characteristics at the MRI phase (2012–2016) for the full Whitehall II Imaging cohort (*N* = 774) and the subsamples selected for the structural (*N* = 542) and perfusion (*N* = 112) MRI analyses. The population means or frequencies from the full cohort were compared with observed sample means or frequencies using 1-sample *t* tests (for means) or chi-squared tests (for frequencies), and the corresponding *p*-values are reported below. MRI, magnetic resonance imaging.
(DOCX)

S2 Table Results of 95% CIs of mediation (5,000× Bootstrapping). CIs excluding 0 are highlighted in bold. CI, confidence interval; FA, fractional anisotropy; M, mediator variable; PWV, pulse wave velocity; RD, radial diffusivity; SF, semantic fluency; VL, verbal learning; X, predictor variable; Y, outcome variable.
(DOCX)

S3 Table Associations of rate of arterial stiffening (ΔPWV) with brain and cognitive outcomes, with and without adjustment for baseline PWV. Model 1: full model as presented in Table 3, adjusting for sex, education, socioeconomic grade, scanner model, baseline age, BMI, MAP, antihypertensive medication, years from Phase 9 to MRI, years from Phase 11 to MRI, and baseline PWV. Model 2 was the same as Model 1 but excluded baseline PWV. Model 3 tested the association with the residuals from the regression of Phase 11 PWV on Phase 9 PWV instead of associations with ΔPWV. Thus, Model 3 did not include ΔPWV and baseline PWV as covariates; however, all the other covariates remained the same. The models show that the overall associations of rate of arterial stiffening with brain/cognition remain similar with and without adjustment for baseline PWV. AD, axial diffusivity; BMI, body mass index; CBF, cerebral blood flow; CI, confidence interval; FA, fractional anisotropy; GM, grey matter, ICV, intracranial volume; MAP, mean arterial pressure; MD, mean diffusivity; MoCA, Montreal Cognitive Assessment; MRI, magnetic resonance imaging; PWV, pulse wave velocity; RD, radial diffusivity, WML, white matter lesion.
(DOCX)

## Abbreviations

AD: axial diffusivity
APOE4: apolipoprotein E4
BMI: body mass index
CBF: cerebral blood flow
CVD: cardiovascular disease
DTI: diffusion tensor imaging
FA: fractional anisotropy
FLAIR: fluid-attenuated inversion recovery
FSL: FMRIB Software Library
FWE: family-wise error
GLM: general linear model
GM: grey matter
HVLT-R: Hopkins Verbal Learning Test–Revised
MAP: mean arterial pressure
MD: mean diffusivity
MoCA: Montreal Cognitive Assessment
MRI: magnetic resonance imaging
pCASL: pseudocontinuous arterial spin labelling
PWV: pulse wave velocity
RD: radial diffusivity
STROBE: Strengthening the Reporting of Observational Studies in Epidemiology
TFCE: threshold-free cluster enhancement
WAIS-IV: Wechsler Adult Intelligence Scale-Fourth Edition
WM: white matter
WML: white matter lesion
