# Supplementary figures and images for: Associations between arterial stiffening and brain structure, perfusion, and cognition in the Whitehall II Imaging Sub-study: A retrospective cohort study
Source: PLoS Med. 2020 Dec 29;17(12):e1003467. doi: 10.1371/journal.pmed.1003467 (PMC7771705; doi:10.1371/journal.pmed.1003467)

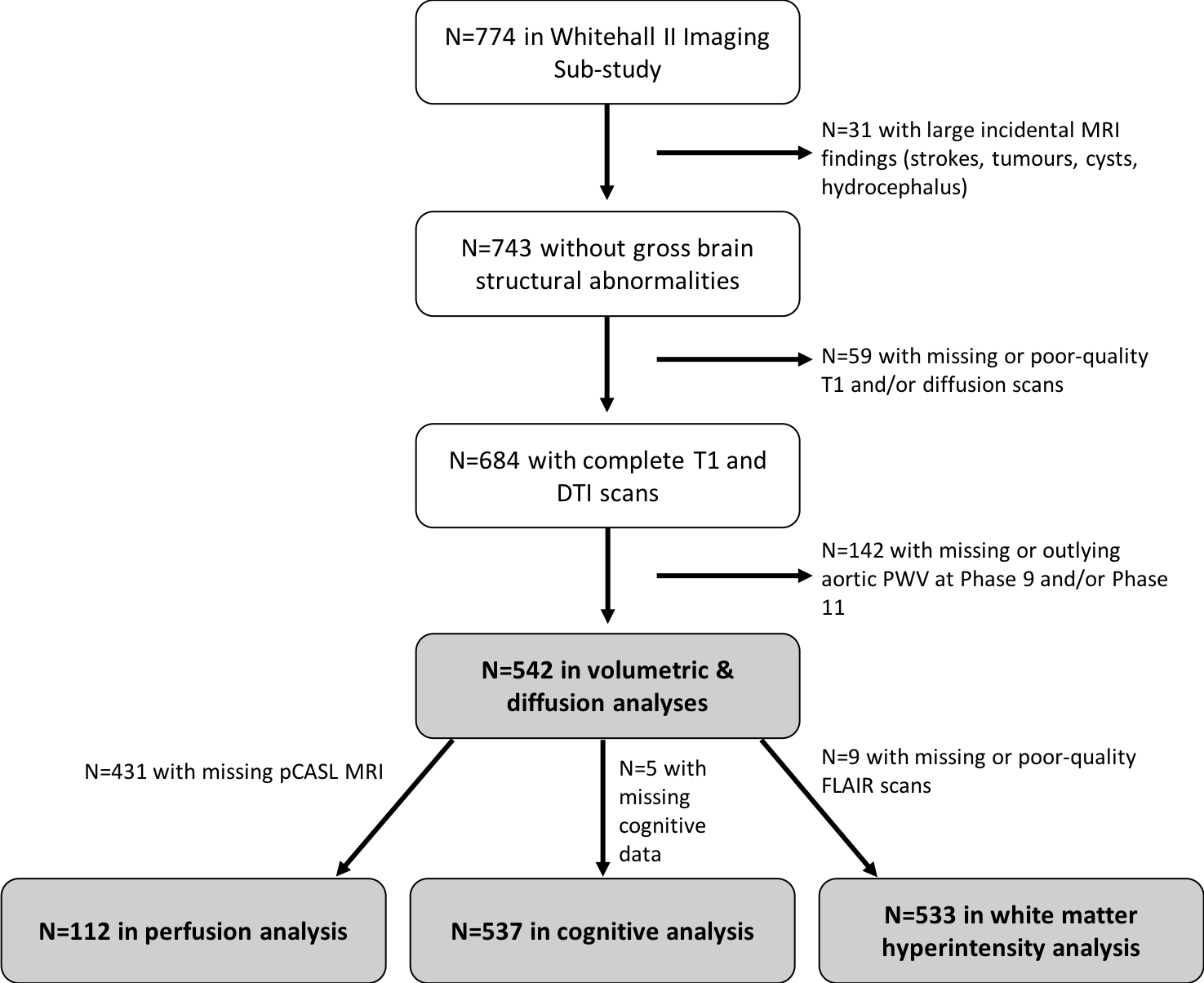

Supplement: S1 Fig — (TIF) [file pmed.1003467.s001.tif]

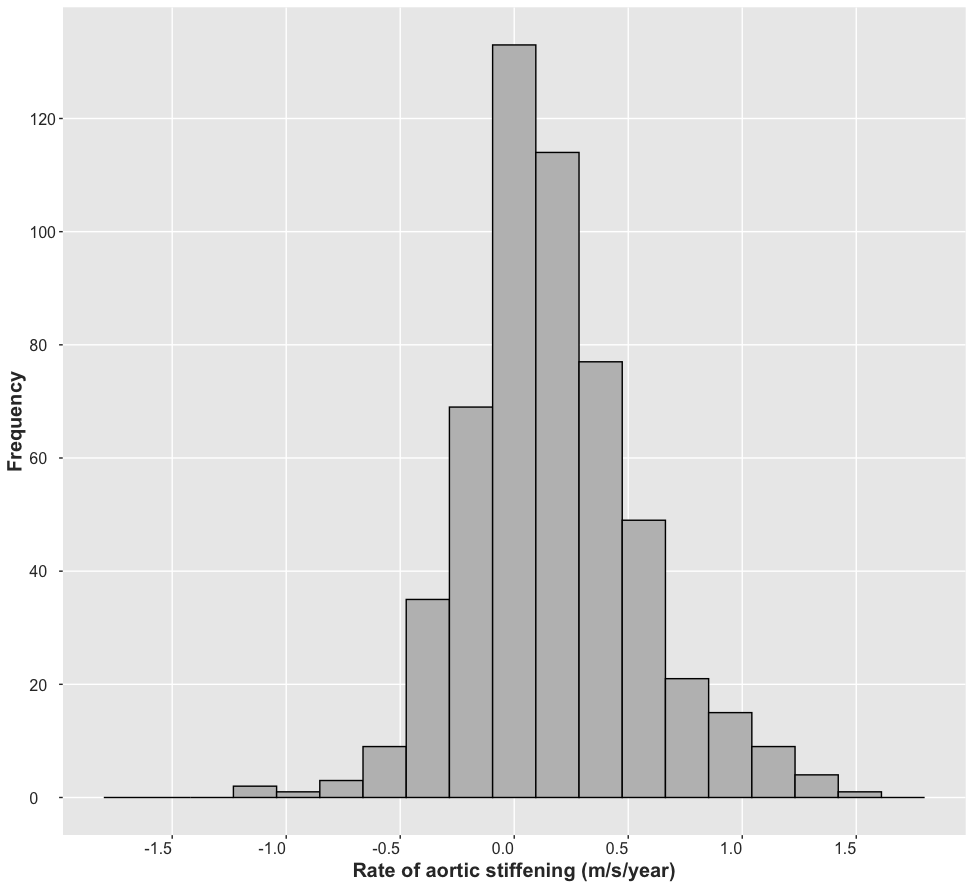

Supplement: S2 Fig — Mean change was 0.2 ± 0.4 m/s per year, ranging from −1.2 to 1.5. (TIF) [file pmed.1003467.s002.tif]
