# Supplementary material for: Associations between arterial stiffening and brain structure, perfusion, and cognition in the Whitehall II Imaging Sub-study: A retrospective cohort study
Source: PLoS Med. 2020 Dec 29;17(12):e1003467. doi: 10.1371/journal.pmed.1003467 (PMC7771705; doi:10.1371/journal.pmed.1003467)
Supplement: S1 Text — MRI, magnetic resonance imaging. (DOCX) [file pmed.1003467.s003.docx]

**S1 Text**

***MRI Image Acquisition***

On the Verio scanner, T1 scans were acquired using a gradient echo sequence (TR = 2530ms, TE= 1.79/3.65/5.51/7.37ms, flip angle = 7°, FOV = 256mm, voxel dimension = 1.0 mm isotropic, acquisition time = 6m12s). Diffusion-weighted images were collected using an echoplanar sequence, with 60 diffusion-weighted directions (*b*-value = 1500 s/mm2), 5 non-diffusion weighted images (*b*-value = 0s/mm2) and one b0 volume in the reversed phase-encoded direction (TR = 8900ms, TE = 91.2ms, FOV= 192 mm, voxel dimension = 2.0mm isotropic). FLAIR images were acquired with the following parameters: TR = 9000ms, TE = 73, voxel dimension=0.9x0.9x3mm^3^, FOV = 220, acquisition time = 4m14s. On the Prisma scanner, T1 scans were acquired using a gradient echo sequence (TR = 1900ms, TE= 3.97ms, flip angle = 8°, FOV = 192mm, voxel dimension = 1.0 mm isotropic, acquisition time = 5m31s). FLAIR (TR = 9000ms, TE = 73, voxel dimension=0.4x0.4x3mm^3^, FOV = 220, acquisition time = 4m14s) and diffusion-weighted images, were matched in protocol except for a change in echo time (TE = 91ms). A multiple postlabeling delay pCASL scan was used to quantify absolute resting CBF (repetition time, 4240 milliseconds; echo time, 13 milliseconds; voxel size, 3.4x 3.4x4.5 mm; flip angle, 90; slice thickness, 4.5 mm; labeling duration, 1400 milliseconds; postlabel delays, 0.25, 0.50, 0.75, 1.0, 1.25, 1.5, and 1.75 seconds) [1]. Two calibration scans (repetition time, 10 000 milliseconds; echo time, 13 milliseconds) were acquired to calibrate the pCASL perfusion-weighted signal via the equilibrium magnetization of blood.

***MRI Image Analysis***

All images were analysed using FMRIB Software Library (FSL) tools.[2]

***T1 scans*** were pre-processed using FSL-ANAT, which performs bias correction, brain extraction, and partial-volume tissue segmentation using the FMRIB Automated Segmentation Tool (FAST) [3]. This was used to extract measures of global grey matter (GM) volume, which was normalised to total intracranial volume and multiplied by 100 (GM%). FSL-VBM (Voxel-based morphometry)[4] was used to generate GM density maps for each subject, which were entered into voxel-wise statistics as described below.

***FLAIR scans*** were automatically segmented using the Brain Intensity AbNormality Classification Algorithm (BIANCA), a fully-automated, supervised method for white matter lesion (WML) detection [5]. Briefly, BIANCA classifies the image voxels based on intensity features (FLAIR, T1 and fractional anisotropy), and spatial features. To avoid scanner-specific biases in these estimates, BIANCA was initially trained with WML masks manually delineated in a sub-sample of individuals scanned on the Prisma (n=24) and Verio (n=24) scanners and an independent sample from the UK Biobank study (n=12). The resulting spatial map represents the probability per voxel of being WML. Voxels exceeding a probability of 0.9 of being WML and located within a white matter mask as described in [5] were included in the final WML map. Total WML volume was normalised to total intracranial volume and multiplied by 100 (WML%).

***Diffusion-weighted scans*** were processed using the FMRIB diffusion toolbox (FDT), which performs motion and eddy current correction with FSL-TOPUP [6]. FA and diffusivity maps were extracted using DTIFit and aligned into standard space using FMRIB's Nonlinear Registration Tool (FNIRT). The mean FA (fractional anisotropy) image was calculated using tract-based spatial statistics (TBSS), and then thinned to create a mean FA skeleton representing the centres of all WM tracts common to the group [7]. This method was repeated for mean, radial and axial diffusivity (MD, RD and AD). Measures of global FA, MD, RD and AD were extracted from the mean skeleton, and the respective subject-specific spatial maps were concatenated and entered into voxel-wise statistics.

***Arterial spin labelling scans*** were processed to derive absolute resting cerebral blood flow (CBF) maps as described previously [8]. We used the Bayesian Inference for Arterial Spin Labeling (BASIL) MRI tool in FSL, which uses a variational Bayes approach to perform a nonlinear fit of the general kinetic model to the pCASL data for all voxels in the brain [9]. BASIL performs head motion and partial-volume corrections, and registers perfusion maps to MNI152 standard space to obtain grey matter CBF maps, which were used in voxel-wise statistics.

***References***

[1] Okell TW, Chappell MA, Kelly ME, Jezzard P. Cerebral blood flow quantification using vessel-encoded arterial spin labeling. J Cereb Blood Flow Metab 2013;33:1716–24. https://doi.org/10.1038/jcbfm.2013.129.

[2] Jenkinson M, Beckmann C., Behrens TE, Woolrich MW, Smith SM. FSL. Neuroimage 2012;62:782–90.

[3] Zhang Y, Brady M, Smith S. Segmentation of brain MR images through a hidden Markov random field model and the expectation-maximization algorithm. IEEE Trans Med Imaging 2001;20:45–57. https://doi.org/10.1109/42.906424.

[4] Douaud G, Smith S, Jenkinson M, Behrens T, Johansen-Berg H, Vickers J, et al. Anatomically related grey and white matter abnormalities in adolescent-onset schizophrenia. Brain 2007;130:2375–86. https://doi.org/10.1093/brain/awm184.

[5] Griffanti L, Jenkinson M, Suri S, Zsoldos E, Mahmood A, Filippini N, et al. Classification and characterization of periventricular and deep white matter hyperintensities on MRI: A study in older adults. Neuroimage 2017. https://doi.org/10.1016/j.neuroimage.2017.03.024.

[6] Behrens TEJ, Woolrich MW, Jenkinson M, Johansen-Berg H, Nunes RG, Clare S, et al. Characterization and Propagation of Uncertainty in Diffusion-Weighted MR Imaging. Magn Reson Med 2003;50:1077–88. https://doi.org/10.1002/mrm.10609.

[7] Smith SM, Jenkinson M, Johansen-Berg H, Rueckert D, Nichols TE, Mackay CE, et al. Tract-based spatial statistics: voxelwise analysis of multi-subject diffusion data. Neuroimage 2006;31:1487–505. https://doi.org/10.1016/j.neuroimage.2006.02.024.

[8] Suri S, Topiwala A, Chappell MA, Okell TW, Zsoldos E, Singh-Manoux A, et al. Association of Midlife Cardiovascular Risk Profiles with Cerebral Perfusion at Older Ages. JAMA Netw Open 2019. https://doi.org/10.1001/jamanetworkopen.2019.5776.

[9] Chappell MA, Groves AR, Whitcher B, Woolrich MW. Variational Bayesian inference for a nonlinear forward model. IEEE Trans Signal Process 2009. https://doi.org/10.1109/TSP.2008.2005752.
