# Supplementary material for: Associations between arterial stiffening and brain structure, perfusion, and cognition in the Whitehall II Imaging Sub-study: A retrospective cohort study
Source: PLoS Med. 2020 Dec 29;17(12):e1003467. doi: 10.1371/journal.pmed.1003467 (PMC7771705; doi:10.1371/journal.pmed.1003467)
Supplement: S2 Text — (DOCX) [file pmed.1003467.s004.docx]

**S2 Text**

**Mediation Analysis**

To test whether the association of Phase 9 PWV on cognitive function at the MRI Phase (verbal learning score, semantic fluency score) was mediated by white matter integrity (FA, radial diffusivity), we used PROCESS Procedure for SPSS Version 3.4 (© Andrew F. Hayes (2018), [www.guilford.com/p/hayes3](http://www.guilford.com/p/hayes3)). Covariates for all analyses were age, mean arterial pressure, treatment with antihypertensives, and BMI during phase 9 and sex, education, socio-economic stratum, scanner model, and years from phase 9 to date of scan.

We used the (Mediation) Model 4 of the software, with pulse wave velocity in phase 9 set as the x-variable, FA, radial diffusivity, respectively, set as mediator (M) variables, and HVLT verbal learning score, semantic fluency score, respectively set as outcome (y-) variables. The number of bootstrap samples for percentile bootstrap confidence intervals was 5000, with the level of confidence for all confidence intervals in output set to 95%.

Pulse wave velocity at Phase 9 predicted performance in both cognitive tests, but none of the MRI mediator variables were independently associated with Phase 9 pulse wave velocity, and consequently none of the indirect (mediated effects) were significant (see S2 Table for results).
