# Supplementary material for: Associations between arterial stiffening and brain structure, perfusion, and cognition in the Whitehall II Imaging Sub-study: A retrospective cohort study
Source: PLoS Med. 2020 Dec 29;17(12):e1003467. doi: 10.1371/journal.pmed.1003467 (PMC7771705; doi:10.1371/journal.pmed.1003467)
Supplement: S1 Table — The population means or frequencies from the full cohort were compared with observed sample means or frequencies using 1-sample t tests (for means) or chi-squared tests (for frequencies), and the corresponding p-values are reported below. MRI, magnetic resonance imaging. (DOCX) [file pmed.1003467.s005.docx]

**S1 Table:** Sample characteristics at the MRI Phase (2012-16) for the full Whitehall II Imaging cohort (N=774), and the sub-samples selected for the structural (N=542) and perfusion (N=112) MRI analyses. The population means or frequencies from the full cohort were compared with observed sample means or frequencies using one sample t-tests (for means) or chi-square tests (for frequencies), and the corresponding p values are reported below.

|  | **Full Whitehall Imaging cohort** | **Sub-sample included in the structural MRI analyses** | **p (structural MRI sample vs full cohort)** | **Sub-sample included in the perfusion MRI analyses** | **p (perfusion MRI sample vs full cohort)** |
| --- | --- | --- | --- | --- | --- |
| N | 774 | 542 |  | 112 |  |
| Age | 69.8 ± 5.2 | 69.8 ± 5.2 | 0.9 | 69.2 ± 4.9 | 0.22 |
| % Female | 22.0 % (150/774) | 18.1% (98/542) | **0.03*** | 13.4% (15/112) | **0.03*** |
| Education | 14.1 ± 3.1 | 14.2 ± 3.0 | 0.15 | 14.1 ± 2.8 | 0.99 |
| Mean Arterial Pressure | 98.8 ± 11.7 | 99.0 ± 11.8 | 0.74 | 97.6 ± 11.4 | 0.25 |
| BMI | 26.2 ± 4.2 | 25.7 ± 3.6 | **<0.001*** | 25.5 ± 3.6 | 0.06 |
| Anti-hypertensive treatment | 66.9% (518/774) | 70.7 % (383/542) | 0.07 | 68.8% (77/112) | 0.69 |
| Current smokers | 3.9% (30/774) | 4.1% (22/542) | 0.94 | 4.5% (5/112) | 0.80 |
| Type II diabetes | 9.0% (70/774) | 6.8% (37/542) | 0.08 | 2.7% (3/112) | **0.02*** |
| Current CVD | 18.1% (140/774) | 18.5% (101/542) | 0.79 | 15.2% (17/112) | 0.44 |
| Current cognitive impairment (MOCA < 26) | 20.7% (160/774) | 17.3 % (94/542) | 0.12 | 22.3% (25/112) | 0.42 |
