# Supplementary material for: Associations between arterial stiffening and brain structure, perfusion, and cognition in the Whitehall II Imaging Sub-study: A retrospective cohort study
Source: PLoS Med. 2020 Dec 29;17(12):e1003467. doi: 10.1371/journal.pmed.1003467 (PMC7771705; doi:10.1371/journal.pmed.1003467)
Supplement: S2 Table — CIs excluding 0 are highlighted in bold. CI, confidence interval; FA, fractional anisotropy; M, mediator variable; PWV, pulse wave velocity; RD, radial diffusivity; SF, semantic fluency; VL, verbal learning; X, predictor variable; Y, outcome variable. (DOCX) [file pmed.1003467.s006.docx]

**S2 Table: Results of 95% confidence intervals of mediation (5000x Bootstrapping).** Confidence intervals excluding 0 are highlighted in bold. Abbreviations: PWV = pulse wave velocity, SF = semantic fluency, VL=verbal learning FA = fractional anisotropy, RD = radial diffusivity, X = predictor variable, Y = outcome variable, M = mediator variable.

|  | X | M | Y | Model  R^2^ | Model  df1/df2 | B_PWV_^†^ | B_M_^†^ | Direct effect (X->Y) | Indirect effect  (X->M->Y) |
| --- | --- | --- | --- | --- | --- | --- | --- | --- | --- |
| Model* 1a | PWV |  | FA** | 0.246 | 10/530 | -0.002;  0.0001 |  |  |  |
|  | PWV | **FA** | **SF** | 0.139 | 11/529 | **-0.739;**  **-0.155** | -0.918;  52.718 | **-0.733;**  **-0.158** | -0.069;  0.004 |
| Model* 1b | PWV |  | RD** | 0.283 | 10/530 | -0.001;  0.029 |  |  |  |
|  | PWV | **RD** | **SF** | 0.137 | 11/529 | **-0.752;**  **-0.160** | -31.00;  4.04 | **-0.737;**  **-0.162** | -0.060;  0.007 |
| Model* 2a | PWV |  | FA** | 0.246 | 10/530 | -0.002;  0.0001 |  |  |  |
|  | PWV | **FA** | **VL** | 0.141 | 11/529 | **-0.601;**  **-0.142** | -1.492;  45.208 | **-0.613;**  **-0.130** | -0.060;  0.005 |
| Model* 2b | PWV |  | RD** | 0.283 | 10/530 | -0.001;  0.003 |  |  |  |
|  | PWV | **RD** | **VL** | 0.139 | 11/529 | **-0.616;**  **-0.144** | -27.89;  3.15 | **-0.616;**  **-0.133** | -0.054;  0.006 |

 *All models are adjusted for age, mean arterial pressure, treatment with antihypertensives, and BMI during phase 9 and sex, education, socio-economic stratum, scanner model, and years from phase 9 to date of scan.

** The first line in each model describes for comparison the regression of the mediator variable (FA, RD) on the independent variable (pulse wave velocity); the second line describes the fully mediated model (pulse wave velocity -> FA, RD -> cognitive function (VL/SF)).

^†^B describes the 95%-confidence intervals of non-standardised regression coefficients, B_PWV_ for pulse wave velocity, B_M_ for the mediator variable (FA, D_rad_) on the dependent (Y)-variable.
