# Supplementary material for: Associations between arterial stiffening and brain structure, perfusion, and cognition in the Whitehall II Imaging Sub-study: A retrospective cohort study
Source: PLoS Med. 2020 Dec 29;17(12):e1003467. doi: 10.1371/journal.pmed.1003467 (PMC7771705; doi:10.1371/journal.pmed.1003467)
Supplement: S3 Table — Model 1: full model as presented in Table 3, adjusting for sex, education, socioeconomic grade, scanner model, baseline age, BMI, MAP, antihypertensive medication, years from Phase 9 to MRI, years from Phase 11 to MRI, and baseline PWV. Model 2 was the same as Model 1 but excluded baseline PWV. Model 3 tested the association with the residuals from the regression of Phase 11 PWV on Phase 9 PWV instead of associations with ΔPWV. Thus, Model 3 did not include ΔPWV and baseline PWV as covariates; however, all the other covariates remained the same. The models show that the overall associations of rate of arterial stiffening with brain/cognition remain similar with and without adjustment for baseline PWV. AD, axial diffusivity; BMI, body mass index; CBF, cerebral blood flow; CI, confidence interval; FA, fractional anisotropy; GM, grey matter, ICV, intracranial volume; MAP, mean arterial pressure; MD, mean diffusivity; MoCA, Montreal Cognitive Assessment; MRI, magnetic resonance imaging; PWV, pulse wave velocity; RD, radial diffusivity, WML, white matter lesion. (DOCX) [file pmed.1003467.s007.docx]

**S3 Table:** Associations of rate of arterial stiffening (∆PWV) with brain and cognitive outcomes, with and without adjustment for baseline PWV. Model 1: full model as presented in Table 3, adjusting for sex, education, socioeconomic grade, scanner model, baseline age, body mass index, mean arterial pressure, antihypertensive medication, years from Phase 9 to MRI, years from Phase 11 to MRI, and baseline PWV. Model 2 was the same as Model 1 but excluded baseline PWV. Model 3 tested the association with the residuals from the regression of Phase 11 PWV on Phase 9 PWV instead of associations with ∆PWV. Thus Model 3 did not include ∆PWV and baseline PWV as covariates, however all the other covariates remained the same. The models show that the overall associations of rate of arterial stiffening with brain/cognition remain similar with and without adjustment for baseline PWV. Abbreviations: PWV: pulse wave velocity, CI: confidence interval, FA: fractional anisotropy, MD: mean diffusivity, RD: radial diffusivity, AD: axial diffusivity, GM: grey matter, ICV: intracranial volume, WML: white matter lesions, CBF: cerebral blood flow, MoCA: Montreal Cognitive Assessment.

|  | **Model 1: Full model for ∆PWV (m/s/y) + covariates** |  | **Model 2: Model 1 excluding baseline PWV** | | **Model 3: Residuals of Phase 11 on Phase 9 PWV + covariates** | |
| --- | --- | --- | --- | --- | --- | --- |
|  | **B [95% CI]** | **p** | **B [95% CI]** | **p** | **B [95% CI]** | **p** |
| **1. Brain Structure (N=542)** | | | | | | |
| **FA [**x 10^-3^] | -5.65 [-9.75, -1.54] | **0.007*** | -4.32 [-8.27, -0.35] | **0.03*** | -1.81 [-3.33, -0.29] | **0.02*** |
| **MD** [x 10^-6^] | 5.66 [-0.06, 11.38] | 0.05 | 4.04 [-1.50, 9.58] | 0.15 | 1.73 [-0.40, 3.85] | 0.11 |
| **RD** [x 10^-6^] | 7.50 [1.36, 13.64] | **0.017** | 5.55 [-0.40, 11.50] | 0.07 | 2.35 [0.07, 4.63] | **0.04*** |
| **AD** [x 10^-6^] | 1.97 [-3.56, 7.50] | 0.48 | 1.02 [-4.31, 6.36] | 0.71 | 0.48 [-1.56, 2.53] | 0.64 |
| **GM (%ICV)** | -0.41 [-1.30, 0.48] | 0.37 | -0.32 [-1.18, 0.53] | 0.46 | -0.13 [-0.46, 0.20] | 0.45 |
| **WML (%ICV, N=533)** | 0.03 [-0.04, 0.10] | 0.39 | 0.03 [-0.04, 0.10] | 0.42 | 0.01 [-0.02, 0.04] | 0.44 |
| **2. Cerebral Blood Flow (CBF, ml/100g/min, N=112)** | | | | | | |
| **Frontal Lobe** | -10.85 [-17.91, -3.79] | **0.003*** | -10.94 [-17.84, -4.03] | **0.002*** | -3.72 [-6.08, -1.36] | **0.002*** |
| **Temporal Lobe** | -7.14 [-12.94, -1.35] | **0.016** | -7.37 [-13.04, -1.69] | **0.01*** | -2.45 [-4.40, -0.51] | **0.014*** |
| **Parietal Lobe** | -12.75 [-21.58, -3.91] | **0.005*** | -12.70 [-21.35, -4.05] | **0.004*** | -4.33 [-7.29, -1.37] | **0.005*** |
| **Occipital Lobe** | -13.30 [-23.87, -2.73] | **0.014** | -13.30 [-23.87, -2.73] | **0.014*** | -4.56 [-8.11, -1.03] | **0.012*** |
| **3. Cognitive Performance (N=537)** | | | | | | |
| **Semantic Fluency** | 0.89 [-0.32, 2.11] | 0.15 | 1.33 [0.16, 2.49] | **0.03*** | 0.41 [-0.03, 0.86] | 0.07 |
| **Verbal Learning** | 0.44 [-0.55, 1.43] | 0.38 | 0.80 [-0.17, 1.76] | 0.10 | 0.25 [-0.12, 0.62] | 0.19 |
| **Delayed Verbal Recall** | 0.18 [-0.40, 0.77] | 0.54 | 0.29 [-0.27, 0.85] | 0.31 | 0.10 [-0.12, 0.31] | 0.39 |
| **Short-term Memory (Digit Span)** | -0.19 [-1.46, 1.08] | 0.77 | -0.15 [-1.37, 1.08] | 0.81 | -0.06 [-0.53, 0.41] | 0.81 |
| **Executive Function (Trail Making)** | -0.01 [-1.11, 0.09] | 0.84 | -0.01 [-0.11, 0.09] | 0.87 | -0.004 [-0.04, 0.03] | 0.83 |
| **Executive Function (Digit Substitution)** | 1.54 [-1.42, 4.50] | 0.31 | 1.78 [-1.07, 4.63] | 0.22 | 0.63 [-0.46, 1.73] | 0.26 |
| **Global Cognition (MoCA)** | -0.08 [-0.57, 0.41] | 0.75 | 0.06 [-0.41 0.53] | 0.81 | 0.002 [-0.18, 0.18] | 0.98 |
